# Decoding the Structural Dynamics and Conformational Alternations of DNA Secondary Structures by Single-Molecule FRET Microspectroscopy

**DOI:** 10.3389/fmolb.2021.725541

**Published:** 2021-09-03

**Authors:** Debolina Bandyopadhyay, Padmaja P. Mishra

**Affiliations:** ^1^ Single-Molecule Biophysics Lab, Chemical Sciences Division, Saha Institute of Nuclear Physics, Kolkata, India; ^2^ HBNI, Mumbai, India

**Keywords:** sm-FRET spectroscopy, DNA secondary structure, DNA hairpin, G-quadruplex, Holliday junctions, i-motif

## Abstract

In addition to the canonical double helix form, DNA is known to be extrapolated into several other secondary structural patterns involving themselves in inter- and intramolecular type hydrogen bonding. The secondary structures of nucleic acids go through several stages of multiple, complex, and interconvertible heterogeneous conformations. The journey of DNA through these conformers has significant importance and has been monitored thoroughly to establish qualitative and quantitative information about the transition between the unfolded, folded, misfolded, and partially folded states. During this structural interconversion, there always exist specific populations of intermediates, which are short-lived or sometimes even do not accumulate within a heterogeneous population and are challenging to characterize using conventional ensemble techniques. The single-molecule FRET(sm-FRET) microspectroscopic method has the advantages to overcome these limitations and monitors biological phenomena transpiring at a measurable high rate and balanced stochastically over time. Thus, tracing the time trajectory of a particular molecule enables direct measurement of the rate constant of each transition step, including the intermediates that are hidden in the ensemble level due to their low concentrations. This review is focused on the advantages of the employment of single-molecule Forster’s resonance energy transfer (sm-FRET), which is worthwhile to access the dynamic architecture and structural transition of various secondary structures that DNA adopts, without letting the donor of one molecule to cross-talk with the acceptor of any other. We have emphasized the studies performed to explore the states of folding and unfolding of several nucleic acid secondary structures, for example, the DNA hairpin, Holliday junction, G-quadruplex, and i-motif.

**GRAPHICAL ABSTRACT:**
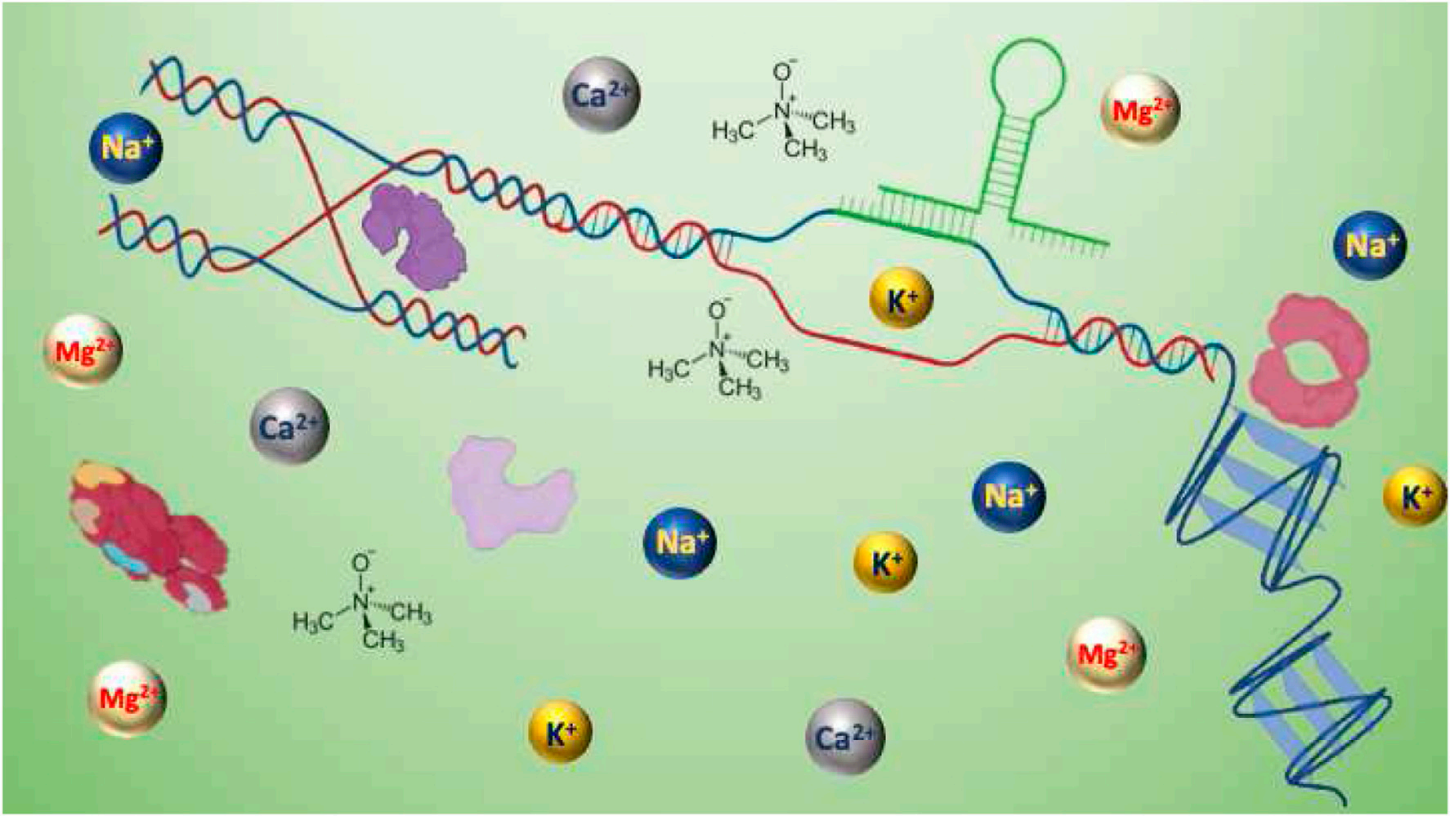


## Introduction

Nucleic acids are known to have a considerable regulatory or functional role at different stages of the majority of the cellular processes. The structural and conformational features of these deoxyribonucleic acid biopolymers provide uniqueness to divergent gene regulatory pathways. Other than adopting the duplex form following the worm-like chain model (Rittman et al., 2009), DNA also acquires several other noncanonical secondary structures either through Watson–Crick type hydrogen bonding or Hoogsteen or reverse Hoogsteen type hydrogen bonds (Spencer, 1959; Zagryadskaya et al., 2003; Nikolova et al., 2011). Polymorphism of the structural aspects of DNA transpires during all-important cellular functions such as replication, transcription, viral genome integration, recombination, DNA supercoiling, genome packaging, vesicular transport, etc. During the accomplishment of the fundamental biomolecular types of machinery, there is the introduction, involvement, or intermission of several nucleic acid secondary structures that come into existence through formation, breakage, or rearrangement of intermolecular or intramolecular hydrogen bonds (Sinden et al., 1998; Wang and Vasquez, 2014). Either these structures need to be self-resolved or they need to be unfastened by the involvement of external factors for the protein of the gene regulatory pathway to come into action (Horwitz and Loeb, 1988; Pearson et al., 1996; Zhao et al., 2010). Under other conditions, the formation of complex intermediates of DNA mediates recognition and binding of proteins for the continuation of smooth gene regulatory processes (Bikard et al., 2010; Brázda et al., 2011; Kaushik et al., 2016). Based on the demand of the cellular process, the continuous transition from folded to unfolded states materializes either in a homogeneous way or heterogeneously, thus by giving rise to higher-order structural polymorphisms, other than being in a single structural state throughout (Ha, 2004; Lee et al., 2005). Additionally, the evolvement of one or multiple intermediate states with partially folded secondary structures might be a requisite for an uninterrupted procession of the biological process. The continuous breathing due to the breaking and formation of the hydrogen bonds at a temperature below the melting point of DNA provides exclusive and microenvironment-specific intrinsic structural features to the secondary structures of nucleic acids (Peyrard et al., 2009; Fei and Ha, 2013; Paul et al., 2017).

**FIGURE 1:**
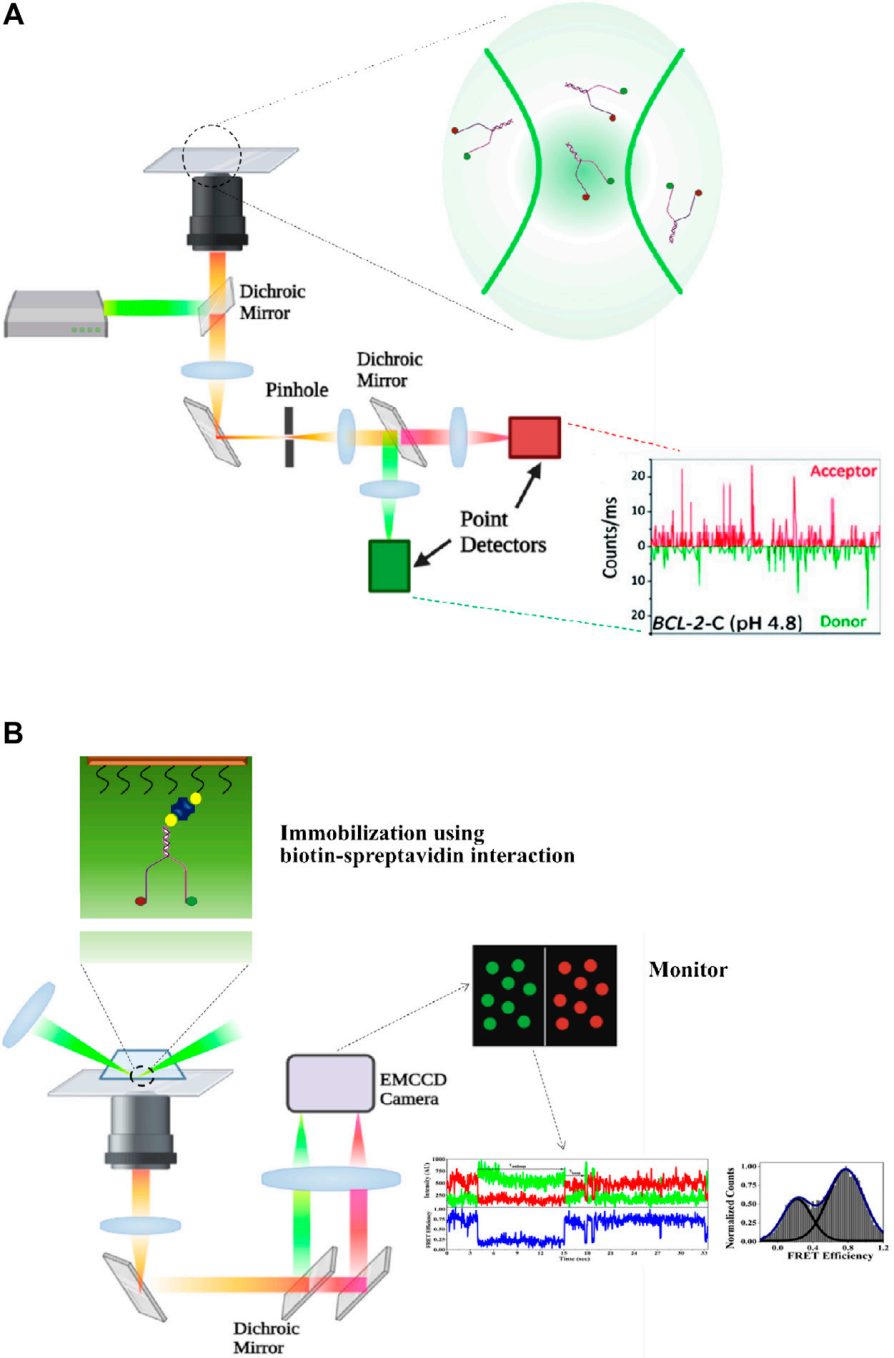
**(A)** Experimental set-up of diffusion type confocal microscopy–based sm-FRET. The molecules of interest are labeled with fluorescent probes acting as the donor and the acceptor. The donor is excited using a laser, and the emitted signals from both the donor and the acceptor are collected and then separated using a dichroic mirror and signals are projected onto photo detectors (APD, Avalanche Photodiode). **(B)** Experimental set-up for prism-type total internal reflection fluorescence microscopy (TIRFM)-based sm-FRET. The sample of interest present in the slide has been expanded to show the site of the labeling of the donor and the acceptor (represented in green and red circles, respectively). The 532-nm laser is used to excite the donor. The emitted rays from both the donor and the acceptor are captured and made to follow two separate pathways using a dichroic mirror. The rays are projected onto a CCD camera and corresponding signals from both the donor and the acceptor are collected (created with BioRender.com).

The binding of proteins with the secondary structure also influences the dynamic response of the system that in turn either promotes further binding of other proteins or causes exclusion for certain proteins to halt additional processivity (Amrane et al., 2005; Alexandrov et al., 2012; Jose et al., 2012). The explicit dynamics of proteins when bound to the secondary nucleic acid structure also play a vital role in the operation of the regulatory system. Proteins either rapidly diffuse over short-length scale units or involve themselves in directional movement along with the DNA. The qualitative and quantitative categorization on the nanometer scale for the understanding and exploration of DNA structures and their motility or the interacting DNA–protein complex has become plausible through the implementation of versatile single-molecule methodologies (Myong et al., 2007; Laurence et al., 2008; Syed et al., 2014). Over the years, development and use of single-molecule techniques have proven to be a sufficient and powerful technology for direct measurement of the heterogeneous distribution in the molecular property, maintaining the phenomenon of monitoring one molecule at a time. Broadly, single-molecule approaches are classified into two branches, force-based methods and non–force-based methods. Atomic force microscopy (AFM) (Ohnesorge and Binnig, 1993), optical tweezers (Ashkin, 1970), and magnetic tweezers (Neuman and Nagy, 2008; Kapanidis and Strick, 2009) are approaches that help to determine the binding forces between individual molecules and accordingly decipher their functions. On the other hand, non–force-based technologies require fluorescence labeling to get a visual perception. Being highly sensitive and having a high spatial and temporal resolution, fluorescence-based detection methods have always been in the limelight. Compared to the other fluorescence-based detection methods (polarization, quenching, and fluorescence correlation spectroscopy) (Deniz et al., 2008), FRET has been considered as the most general, adoptable, and indispensable tool to extract the physical behavior of biological phenomena (Ha et al., 1996). Through sm-FRET, it has become possible to extensively capture the structural intermediates and draw valuable fathomable information about the transition dynamics and energy of the state (Lerner et al., 2013). sm-FRET has made it possible to investigate the structural dynamics of biomolecules at a remarkable resolution and sensitivity by extracting the structural transposition of bimolecular complexes passing through multiple conformational alternations (Ha, 2001a; Hohng et al., 2004a; McKinney et al., 2006). An impeccable combination of spectroscopy and microscopy, the popularity of sm-FRET microspectroscopy comes from its competence to report on dynamic, either intra- or intermolecular, interactions in real time. The biomolecules need to be site-specifically labeled with distinct fluorophores acting as the donor (D) and the acceptor (A), having substantial spectral overlap. As energy transfer between a single donor and acceptor is monitored, technically, this method is also referred to as single-pair FRET (Lu, 2014). The degree of non-radiative energy transfer between the donor and the acceptor is guided by the extent of the proximal distance between them, and hence sm-FRET is considered as an effective one-dimensional ‘spectroscopic ruler’ in the range of 1–10 nm (Ha, 2001b; Lu, 2005; Yu et al., 2006). Since the distance sensitivity of this method lies in the range of the length scale of conformational changes during biological phenomena, it is advantageous to explore molecular conformational dynamics of the system either in diffused or in immobilized form. In diffusion-based sm-FRET methods, mostly the molecules are imaged using a confocal microscope, and the intensity of the donor and the acceptor is captured using an Avalanche photodiode (APD) (Ha et al., 1996). For smooth and accurate data acquisition, the molecular concentration is kept very low so that there is the presence of one molecule at a time in the small confocal volume which in turn further ensures a small observation volume and hence a high signal-to-noise ratio (Figure 1A). For monitoring FRET by immobilization of the system of interest, the typical strongest biotin–streptavidin interaction (Green, 1975) is used for surface attachment of the labeled molecules and keeping them away from each other at the minimum distance of the diffraction limit (Sasmal et al., 2016). Here, the phenomenon of the high signal-to-noise ratio is maintained using total internal reflection fluorescence microscopy (TIRFM)-based detection methods where excitation through evanescent waves is generated at the point of total internal reflection. This also ensures restricted illumination with a general penetration depth of 100–200 nm and that leads to the interference of the minimum possible background (Figure 1B) (Ha, 2001b; Roy et al., 2008). The tracks of the individual molecules those are devoid of cross-linking within themselves is captured using an electron multiplying charged coupled device (emCCD) camera with high time resolution. The spatial resolution in both methods is fixed and is equivalent to the point spread function of a diffraction-limited spot, which is 250 nm (Roy et al., 2008). In this way, the phenomenon of ensemble averaging is resolved, and the details of the respective modulated individual states of every individual molecule are revealed. The detailed knowledge about the conformational dynamics and their variable degree of stability in different microenvironments gained using sm-FRET as a tool acts as a cue for extracting the mechanical processivity, biochemical functions, and physical properties of the system that includes the speed, step size, and directionality. Undeniably, the evolvement of sm-FRET is paving the way toward the new area of ‘dynamic structural biology’ (Ha et al., 2012; Mohapatra et al., 2019; Lerner et al., 2021).

In this review, we have focused on documenting the possible intricate details of the dynamic conformers of four different secondary structures of DNA, *viz*., the DNA hairpin, Holliday junction, G-quadruplex, and i-motifs collocated by employing sm-FRET as the preeminent tool. The assortment of distinct conformational features and the transition during different stages of the biomolecular mechanisms and while interacting with associated proteins have been reported here.

### Types of Hydrogen Bonding in DNA Structures

The polymer of deoxyribonucleic acid is manifested into a highly organized regular form through the involvement of various kinds of hydrogen bonds between the constituent units. The regular right-handed double-helical B-form DNA is formed due to Watson–Crick type hydrogen bonds within the subunits, where both the purine and pyrimidine bases are in *anti* conformation. Upon 180^º^ rotation of the purine base along with the glycosidic bond, the *syn* conformation is attained by the purine base, giving rise to the formation of Hoogsteen type hydrogen bonds. In the cellular system, during the high level of organization and continuation of molecular mechanisms, there is the introduction of negative supercoiling, and for relaxing these alterations, various DNA secondary structures arise. Either Watson–Crick type hydrogen bonding or Hoogsteen or reverse Hoogsteen type interactions stabilize these secondary structures. Sometimes these form within certain conserved motifs of the genome, and in other cases, random sequences extrapolate to give rise to various secondary structures, as described below.

### DNA Hairpin

Hairpin loops, short secondary structural folds formed by both DNA and RNA, mostly appear as intermediates during many biological processes. These loops form in single-stranded nucleic acids and consist of a base-paired stem and a loop sequence with unpaired nucleotide bases. The formation of the DNA hairpin takes place due to intra-strand base pairing between the inverted repeats (IRs) of a single strand of DNA (ssDNA). The intermediate events during the commencement of several cellular processes wherever there is an introduction of a single-stranded palindromic DNA structure witness the formation of the DNA hairpin. During the progression of replication, the site of the DNA at the origin of replication requires melting of large regions, resulting in long single-stranded DNA. Also, the gaps between two noncontinuous nascent Okazaki fragments give rise to single-stranded DNA. This single-stranded DNA formed during the replication process and that carries the sequence of inverted repeats eventually hybridizes to the form of a hairpin either with a single loop or with multiple loops (found during viral genome replication) (Enemark and Joshua-Tor, 2006). Additionally, unzipping of DNA at the region of the transcription bubble results in the creation of unstable hairpins that necessitate being resolved for the advancement of the process of the central dogma. Also, during the process of conjugation, transfer of the bacterial genome occurs *via* the introduction of single-stranded DNA intermediates in the donor bacterium by the action of relaxase protein. Correspondingly, there is a brief introduction of single-stranded DNAs during the process of DNA repair and recombination. A continuous stretch of polypurine repeats in the regulatory region of the genome, for example, in the introns or promoters, is extrapolated into a noncanonical Watson–Crick hairpin (Faucon et al., 1996; Goñi et al., 2006). From the cellular context, it is clear that physiologically, DNA hairpins need to be resolved to carry forward the process of gene regulation and thus are dynamically very flexible. Other than having roles in biological intermediates, DNA hairpins also act as biosensors and play major roles in DNA nanotechnology (Tomov et al., 2013). The formation and stability of DNA hairpins have been analyzed through gel-based assays, NMR, and CD spectroscopy (Summers et al., 1985). Also, several calorimetric assays have been conducted where the degree of stability is accessed *via* the melting temperature of the hairpin (Scheffler et al., 1970; Wemmer et al., 1985; Senior et al., 1988). However, the distribution of molecules at various structural forms and deeper information about transition dynamics remain missing through these techniques and thus single-molecule investigation fills the gap by providing crucial details about the structural dynamicity of the system.

**FIGURE 2:**
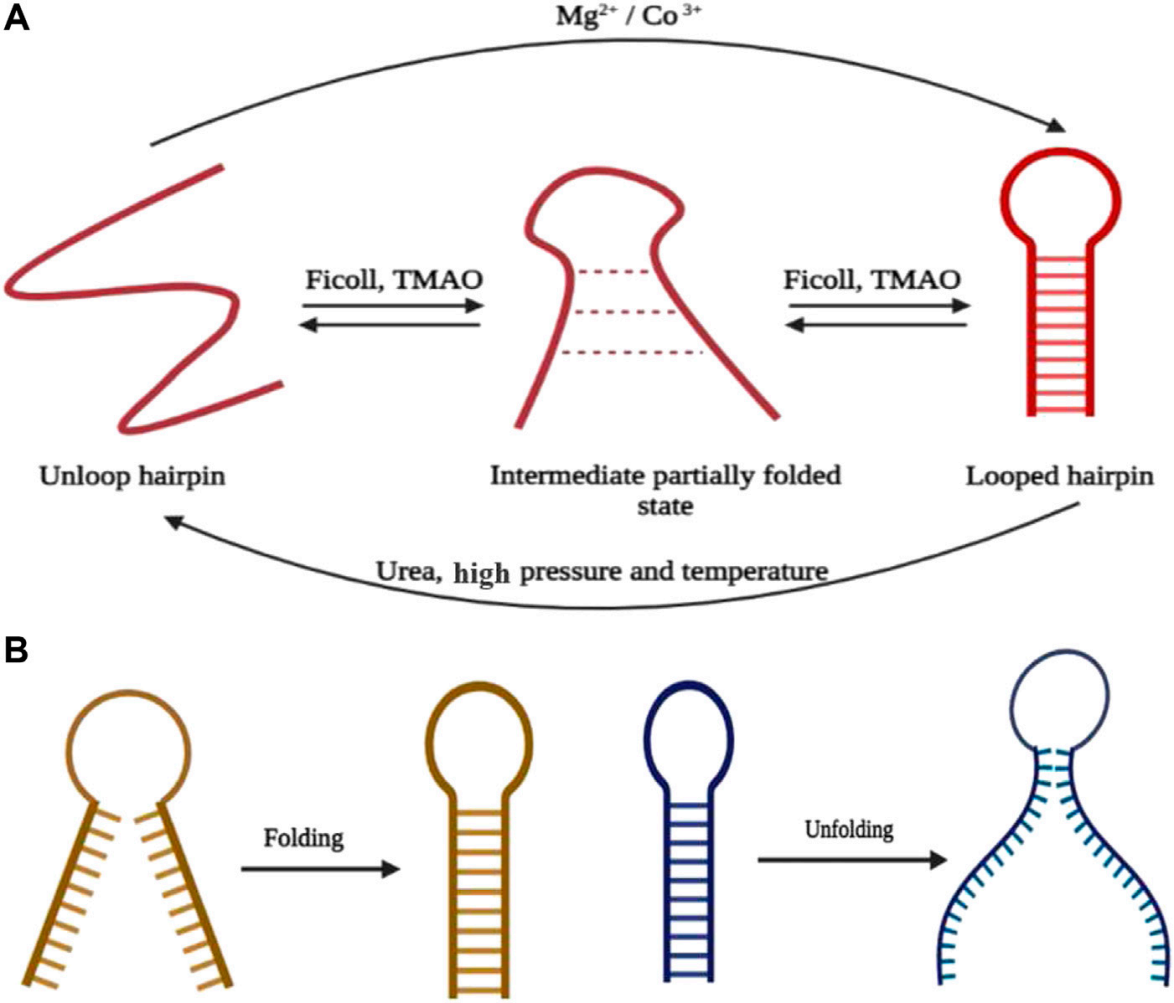
**(A)** Summary of hairpin formation going through the intermediate state in the presence of different ions, cosolutes, and physical environmental conditions. **(B)** Mechanism of folding and unfolding of the hairpin through end-to-end collapse and unzipping from the end, respectively (created with BioRender.com).

The sm-FRET technique has been successfully employed to invigilate and resolve the degree of unsynchronized fluctuations throughout the conformational energy landscape of the nucleic acid secondary structure with the minimum possible dynamical averaging. Likewise, both qualitative and quantitative features regarding the partially folded intermediate state of any kind of vigorous secondary structure of DNA can be extracted more vividly through molecule-by-molecule analysis. Several sm-FRET investigations on DNA hairpins having either poly adenine-repeats or several random nucleotides at the loop region and variable sticky nucleotides at the stem region having the ability to be hybridized have been carried out to develop an improved understanding of the hairpin dynamics. It has been observed through FRET efficiency analysis that the individual hairpin molecules, besides being in the low (E_FRET_ = 0.14) and high FRET states (E_FRET_ = 0.85) (corresponding to folded and unfolded conformation, respectively) (Santoso et al., 2010), also acquire a bridging conformation in the intermediate FRET efficiency region (E_FRET_ = 0.51) amidst the two extreme states (Amrane et al., 2005; Tsukanov et al., 2013). The conformation in the bridge region is resolved into more multiple states if the hairpin has multiple bulges and loops (Chen et al., 2014). The degree of fluctuation between the open and closed-loop states of the hairpin is primarily governed by the sequence of nucleotides in the stem region and the counterion concentration (Mitchell et al., 2018), as positively charged cations minimize repulsion between the backbone phosphate anions (Woodside et al., 2006). The onset of hybridization *via* Watson–Crick bonds within the inverted repeats of the stem region of the DNA hairpin starts with the addition of cations, be it monovalent, divalent, or trivalent ions. However, the degree of stability of the DNA hairpin depends upon the charge density of the salt and hence increases with increments in the valency of the cations. While the minimum monovalent cationic concentration required to promote closed hairpin conformation increases with a decrease in G-C content in the stem region, there is the commencement of closed hairpin conformation even at a meager concentration of divalent or trivalent cations. The relative height of the peak of the FRET efficiency histogram of the open state decreases eventually, with regard to the peak of the closed state upon increments in counterion concentration. When the stem length increases from 70 to 110 bp, the length of dsDNA in the stem region governs the concentration of cationic charges required to push the population towards the folded state following the trend of declination in the strength of monovalent ion concentration (Mustafa et al., 2018).

sm-FRET is also successful in monitoring the dependence of high pressure and temperature on the closed hairpin population and it has been found that the elevated degree of physical properties in the environment offers an inclination toward the open state conformation in the presence of only monovalent cationic salts in the background. Nevertheless, buffers supplemented with di- or trivalent cations (e.g., Mg^2+^ or Co^3+^) impose counteraction to the destabilizing effect of raised pressure. Additionally, hairpin-stabilizing complements such as trimethylamine-N-oxide (TMAO) in the buffer nullifies the hydrogen bond weakening the effect of pressure and temperature. The degree of stabilization is in good agreement with the increase in the concentration of TMAO (Patra et al., 2019). Similar effects have been found in the presence of molecular crowder like 20% by weight Ficoll. Conversely, the fraction of molecules in open hairpin conformation transcends the fraction residing in the closed form when exposed to the denaturing osmolyte urea, and this holds valid for DNA hairpins with multiple loops (Chen et al., 2014). A fraction of molecules transitioning between the two poles of folded and unfolded states pass through the semi-folded or intermediate conformation where there is incomplete base-pairing in the stem region. An influx of this population is clear when there is a lower cationic charge or stabilizing osmolytes such as TMAO in the medium or if the experiments are executed at a temperature higher than ambient values. In the presence of urea, the conformational landscape becomes more complex with all molecules exhibiting three-state behavior. Also, the folded state of the hairpin itself displays the bimodal distribution of FRET efficiencies, and there is an exhibition of two more states right in the closed conformation of the hairpin. However, an increase in the concentration of urea leads the population to shift toward a fully open state. The diversity of the conformational landscape of the hairpin gains more momentum when it is subjected to crowding agents such as Ficoll, portraying a bimodal distribution both at the looped and unlooped states (Figure 2A). The above sets of experiments thus indicate that the addition of urea or crowding agents as co-solutes causes the dynamical states to slow down. So, it helps in decoding multiple intermediate conformational states, paving the way toward the folding and unfolding mechanism of the DNA hairpin (55). Hairpin dynamics has also been evaluated in the presence of gold nano-antennas (GNAs), and there is an enhancement of the fluorescent signal of Cy3 (as donor) and Cy5 (as acceptor) in the presence of GNAs and a drop in the FRET efficiency value of both the closed and open states (Hu et al., 2018). The hidden Markov model (HMM) is widely followed to evaluate the dynamic transition rates of the folded to the unfolded state and the reverse process, too (McKinney et al., 2006; Lee, 2009).

The reduction in stem G-C content has a direct effect in increasing the opening rate of the hairpin and the population residing in the transition state of the partially folded configuration. Although the influence of the stem sequence on the rate of hairpin closure is insignificant, the closing rate accelerates with the reduction in intramolecular columbic repulsion within the DNA backbone (Tsukanov et al., 2013). The existence of divalent cations further increases the folding rate (from 7.5 to 13.3 s^−1^) of the hairpin and since in a contented neutral medium, unzipping of the hairpin stem is cumbersome, the opening rate illustrates a decline in magnitude. Henceforth, it can be concluded that an increase in cationic charge in the medium causes the geometrically compact state to be more stable. Alternatively, TMAO acting as the stabilizing osmolyte intensifies the folding rate (23.2 s^−1^), while the unfolding rate remains unaffected with respect to the solution where it is absent. Similar effects have been found in the presence of Ficoll acting as a crowding agent. In agreement with the chemical property, a denaturing agent such as urea increases the unfolding rate and leads to a drop in the folding rate of the hairpin. The results of several sm-FRET and related single-molecule studies have shown that the hairpin folding process, either with a single loop or with multiple loops, follows the mechanism of “end-to-end collapse” (Figure 2B) (Ma et al., 2007) of hydrogen bonds. However, the unfolding of the bonds opened like a zipper from one end and proceeded toward the other end, termed as “untwisting and peeling” (Figure 2B) (Andreatta et al., 2006; Wong and Pettitt, 2008). The point of fraying is being acted upon by either of the ends (Andreatta et al., 2006) in case of the single-looped DNA hairpin and the intermediatory bulges in DNA hairpins having multiple loops (Chen et al., 2014).

Evaluation of noncanonical hairpins, having reverse Hoogsteen bonds in the stem region within equivalent polypurine residues, illustrates a nonmonotonic relation with increasing concentration of Mg^2+^ ions. Dominance in the population of the partially folded or poised state at near-physiological concentration indicates involvement of either rapid unzipping and reforming of the reverse Hoogsteen bonds or detention in the transient state causing the hairpin to fold or unfold depending upon the gene regulatory demand of the system. Also, the hairpin goes through a partially open state when subjected to a complementary polypyrimidine strand for triplex formation (Bandyopadhyay and Mishra, 2020). The highly sensitive and quantitative output of sm-FRET studies has been considered in designing biosensors where interconvertible hairpin-based sensors (iHaBSs) were proved to act as multiplex sensors based on the density of population in each FRET state. The fluorophore tags are being placed at a position where hairpin formation would cause an increase in FRET efficiency. The hairpin-forming DNA sequence is hybridized using a probe, and the addition of the target sequence causes displacement of the probe, leading to the hairpin formation and, as a result, an increment in the population in the high FRET state. The sensitivity, specificity, and ability to detect multiple targets have been demonstrated by the appearance of the population in the high FRET state. It was found that in response to different targets, three different FRET states have occurred without any false-positive results, and whenever there is a mutation in the target sequence, the population shifts toward the low FRET state (Kaur et al., 2019).

### Holliday Junction

**FIGURE 3:**
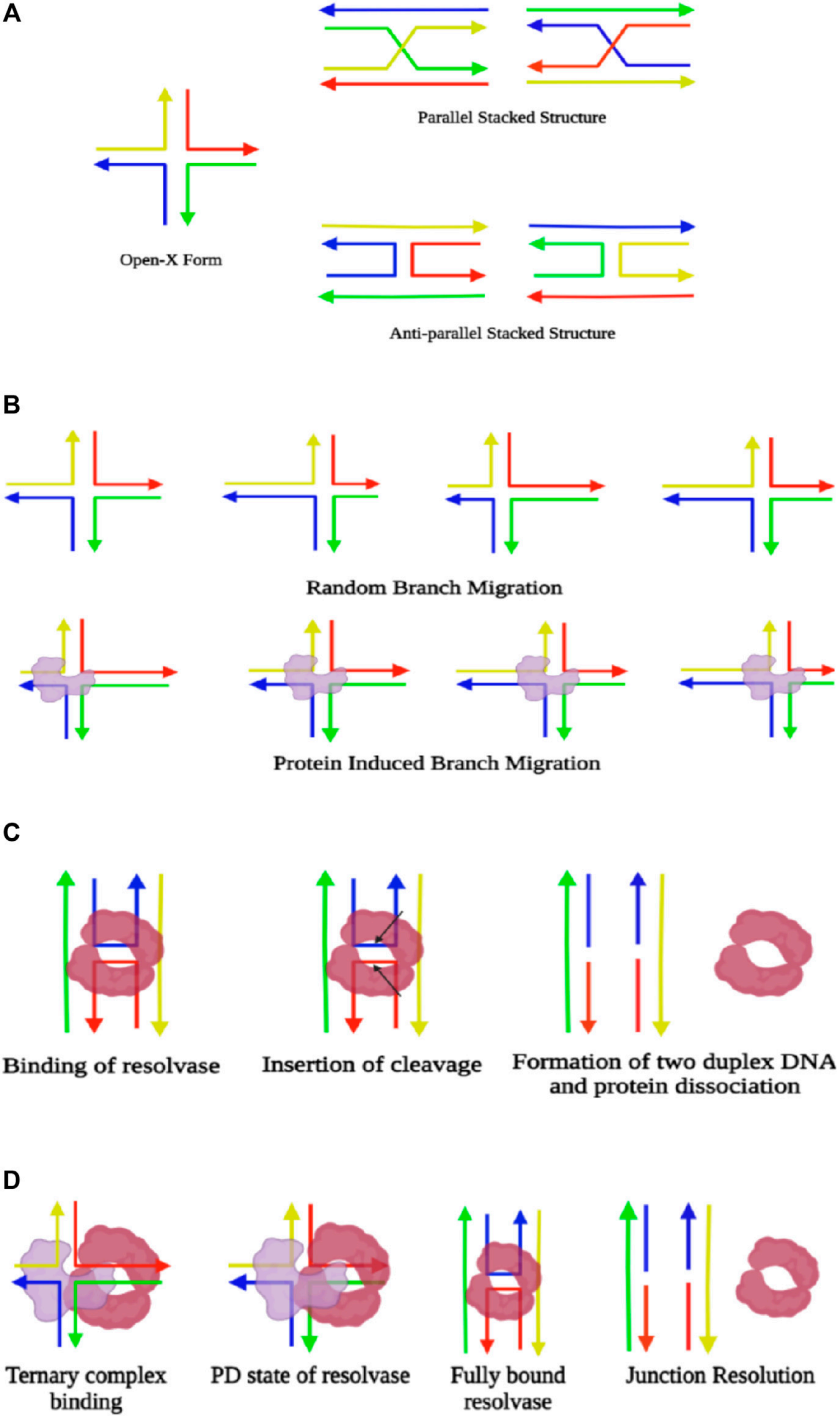
**(A)** Illustration of different forms of Holliday junctions. **(B)** Schematic representation of branch migration, where there is random locomotion of the junction during spontaneous branch migration. However, the presence of protein in the junction (colored in blue) leads to directional branch migration. **(C)** Representation of junction resolution through enzymes. Resolvases (colored in maroon) bind the dimeric form at the junction and insert two symmetrical cleavages (indicated by the arrows), which fixes the four-way junction and gives rise to two duplexes, followed by the dissociation of the protein. **(D)** Demonstration of simultaneous branch migration and Holliday junction resolution. The resolvase and branch migratory enzyme initially form a ternary complex and bind at the junction where the resolvase remains in a partially dissociated state (PD state). Upon branch migration up to the required cleavage site, the resolvase converts itself to a fully bound state and induces the desired nicks for junction resolution (created with BioRender.com).

Holliday junctions (HJs) can be primarily described as a DNA branch-point with four double-stranded DNA complexes joined in the center acting as a critical key intermediate for several cellular processes like meiotic homologous recombination, fork reversal, double-stranded break repair of DNA, processing of stalled replication, and viral genome integration (Holliday, 1964). Structural classification fundamentally divides this into two forms, *viz.*, the extended open-X conformation and stacked-X structure. In open-X or the extended form, the four arms of the double-stranded DNA point toward the four corners of a square, exhibiting four-fold symmetry and utilizing maximum repulsion between the phosphate groups when they are unshielded in the presence of low salt. With the introduction of multivalent counterions in the medium (Panyutin et al., 1995), each pair of DNA is coaxially stacked on top of each other, giving rise to a more stable two-fold symmetrical structure, termed as the stacked-X conformation (Lilley, 2000). Stacked conformations are further diversified into two forms, namely, parallel (Sigal and Alberts, 1972) and antiparallel, based on the nature of the polarity of the helices stacked over one another, out of which the antiparallel (Murchie et al., 1989; Clegg et al., 1992; Eichman et al., 2000) form is topologically rigid. In the parallel form, the polarity of the continuous strand is similar, whereas the antiparallel type shows the opposite polarity in the continuous strand (Nowakowski et al., 1999; Ortiz-Lombardía et al., 1999). The antiparallel stacked structure is further diversified into two isomeric conformations, iso I and iso II, classified based on alternative stacking of different arms of the junction (Figure 3A).

Labeling with the donor and acceptor FRET pairs either at the end or at a distinct place of the adjacent helices of the Holliday junction, sm-FRET has been successfully used for capturing and distinguishing the two isomers exhibiting two explicitly different FRET states. Furthermore, there is frequent cation-dependent interconversion between the two isomers of the antiparallel stacked conformation. sm-FRET and small-angle X-ray scattering (SAXS) (Zettl et al., 2020) have verified the existence of an open-X structure through which the isomers pass during the interconversion. The dependency of interconversion on Mg^2+^ is inversely related, as the rate decreases with an increase in the Mg^2+^ concentration by diminishing the dynamicity of the system and results in a less-visited open form at elevated Mg^2+^ levels. Careful evaluation of results obtained from sm-FRET experiments carried out in the presence of high Mg^2+^ ionic concentration (10 and 50 mM) reflects a strong bias toward the iso II state for the HJs that consist of both AT and GC base pairs, with a higher transition rate in 10 mM Mg^2+^ than in the presence of 50 mM Mg^2+^. However, when the junction consists of explicitly GC base pairs, both the isoforms are equally populated (McKinney et al., 2003). Besides, reducing the Mg^2+^ concentration to 150 µM exhibits an intermediate FRET state throughout, indicating a faster transition timescale between the isomeric states which are indistinguishable through sm-FRET experiments (Zettl et al., 2020). However, existence in the parallel form is so brief that it has not been possible to capture the state, neither utilizing ensemble nor single-molecule techniques, even in a wide range of Mg^2+^ ionic concentrations (Murchie et al., 1989; Duckett et al., 1988; Joo et al., 2004). Stability of the stacked conformer in the presence of monovalent cations only and while supplemented with divalent counterions has been evaluated, and it has been seen that only Na^+^ ions have an almost similar stabilizing effect to that of Mg^2+^, except the fact that a much higher concentration of the monovalent ions is required to exhibit similar behavior. Moreover, the influence of increasing Na^+^ ions, while Mg^2+^ is in the background, is nonmonotonic with a reduction in junction stability up to a particular concentration. Beyond that concentration, the conformer transition rate decreases, promoting more stability to the junction. This is possibly due to the screening effect of the Na^+^ that reduces the interaction between Mg^2+^ and the junction, whereas at a higher concentration, Na^+^ ions show a similar effect to that of Mg^2+^ ions. Similarly, the presence of Na^+^ ions in the background was found to increase the transition rates for every concentration of Mg^2+^ ions due to the screening of electrostatic interactions between Mg^2+^ ions and phosphates. Although, the stacked conformation is very compact, as has been validated through several single-molecule studies, the transition from stacked to the open-square planar form has to cross a low activation energy barrier that allows these two arrangements to be interchangeable (McKinney et al., 2003; McKinney et al., 2005). Holliday junctions need to be processed back into double-stranded recombinant DNA and that occurs through branch migration and resolution of the junction. In eukaryotes, other than resolution, there is another alternative process called dissolution where the junctions are pushed by Bloom’s helicase, followed by decatenation by topoisomerase (Manthei et al., 2013).

Branch migration is the phenomenon that involves the movement of the cross-over junction along with the DNA due to stepwise exchange of base-pairing partners by their sequence homology, either in a unidirectional or a bidirectional way (Constantinou et al., 2001). It is facilitated by the RuvAB complex carrying helicase activity in an ATP-dependent manner in *E. coli*. RuvA is a homotetramer that initially binds to the junction. Then this recruits the RuvB hexameric ring and that binds in an opposite orientation across both sides of the RuvA-bound Holliday junction. The sm-FRET analysis has shown that binding of RuvA with the Holliday junction causes clamping of the protein to the extended open-X structure and halts the conformational switching. Thus, the continuous progression of branch migration is facilitated in the presence of RuvA without any pause (Gibbs and Dhakal, 2018). The free energy change upon switching from stacked to open conformation increases by 2.5 folds when the Holliday junction is subjected to binding with the RuvA protein. Titration with Mg^2+^ ions confirms the interaction of RuvA with the Holliday junction to be electrostatic, as it is disrupted with an increase in Mg^2+^ concentration in the medium since it shields the DNA phosphate and loosens the interaction of the DNA and protein (Gibbs and Dhakal, 2018). While *in vivo*, promotion of branch migration is purely an ATP-driven process. However, it has been observed through several single-molecule studies that being an isoenergetic process with an unaltered number of hydrogen bonds, both unidirectional and bidirectional branch migration occurs spontaneously whenever there is occasional switching to the open-X conformer from the stacked-X state.

It has also been observed that irrespective of their property of branch migration acquiring the stable stacked conformer, all junctions experience occasional switching toward the open extended state even in the presence of high Mg^2+^ ionic concentration (Hohng et al., 2004b). Also, there is no experimental evidence of the existence of a parallel form of the Holliday junction in both types having migratable and non-migratable cross-over regions. Thus, it has been hypothesized that the parallel form is not important even for gene regulatory mechanisms and genetic recombination unless stabilized by proteins (Hohng et al., 2004b). Rearrangement of base pairs during branch migration is a spontaneous stochastic process if not guided by any protein and thus, is described as a one-dimensional random walk process where hoping occurs over multiple base pairs as long as there is homology (Figure 3B). Otherwise, branch migration is inhibited if there is even a single base-pair heterology (Okamoto and Sako, 2016). Henceforth, the two major energy barriers for consequent branch migration are junction unfolding during base-pair rearrangement. Time and energy specificity for junction unzipping is highly sequence-dependent; therefore, the presence of GC pairs in the cross-over point needs to overcome larger barriers than AT base pairs. Also, the unprompted transition from stacked-X to open-X conformers is delayed for the presence of GC base pairs at the cross-over point. A high concentration of divalent salt reduces stacked conformer interconversion, eventually diminishing the occurrence of the open-X structure that results in the reduction of the rate of spontaneous branch migration by 1,000 folds (Johnson and Symington, 1993; Panyutin and Hsieh, 1994). Additionally, spontaneous branch migration is not incessant in the absence of any protein. Rather, the junction transits many times to stacked conformations due to the splitting of the base pairs during extended migration. The activation enthalpy of branch migration being abundantly higher than that of conformer exchange, it results in a pause in branch migration progression. Even the conformer transition rates are much higher than spontaneous branch migration rates under equivalent conditions (McKinney et al., 2003).

The catalytic branch migration process through the ring of helicases has been precisely explored using sm-FRET studies, and it has been observed that instead of facilitating the melting of DNA base pairs by pulling the DNA through the central core, the helicase freezes the junction dynamics by arresting it at open-X conformation. This process is theoretically termed the ‘Brownian ratchet model’ (Rasnik et al., 2008). Due to the introduction of steric hindrance by the proteins, the open-X conformation remains stable, and no conformational transition occurs toward the stacked-X structure even in the presence of high divalent salt. While the helicase clamps the open-X conformation of the HJ, the junction undergoes branch migration spontaneously in a unidirectional manner as the movement of the protein toward the branch point prevents backward migration (Figure 3B). Thus, the presence of RuvAB or other branch-migrating enzymes enables large-scale, quick, and directional junction transition. Nevertheless, *in vivo*, there are shreds of evidence of spontaneous branch migration being almost ten times faster because of the tendency to reach stable branch points (McKinney et al., 2005).

The next step of the process is the resolution of the junction to allow separation of recombinant duplexes, a critical step during homologous recombination, catalyzed by specific junction-resolving enzymes. In prokaryotes, RuvAB recruits RuvC resolvase to form a complex termed as RuvABC “resolvasome” (Constantinou et al., 2001). The binding of junction-resolving enzymes occurs in the dimeric form. It has been hypothesized that before binding to the HJ, the enzyme binds with a duplex DNA and slides or hops along with it until it finds a junction. Binding with the junction introduces symmetrically paired bilateral cleavage, resulting in the formation of two nicked DNA species, followed by the dissociation of the protein (Figure 3C). The most common junction resolvase RuvC exists as a homodimer, thus binding the junction in a dimeric state, and consequently induces two incisions, out of which the second incision is ∼150-fold faster than the first one (Fogg and Lilley, 2000). On the other hand, its eukaryotic counterpart, GEN1, captures the dynamically interchanging conformer of the Holliday junction in a monomeric state. Stable binding of the GEN1 monomer leads to the primary molding of the Holliday junction and further catalyzes the forward reaction toward dimer formation. Once the junction is exposed to the dimeric protein, the introduction of two consequent cleavages takes place, promoting the process of junction resolution (Sobhy et al., 2019). It has been found that sequence-specific enzymes that fall into the category of recombinase, integrase, or DNA repair enzymes prefer to bind at open-X conformation and promote branch migration. On the other hand, sequence-independent enzymes such as resolvases bind to stacked-X structures, where migration of the junction is not essential (Fogg et al., 2001; Khuu et al., 2006). Even though electrostatic interaction plays a key role in the binding of enzymes with the junction, the presence of inhibitory aromatic peptides accelerates the dissociation of catalytic enzymes. The analysis of the dynamic nature of the junction upon hexapeptide binding portrays that influence of Mg^2+^ ions on the junction fluctuation, which is a sharp drop for both AT- and GC-based junctions. Also, the FRET states due to peptide binding depict the acquirement of a range of configurations, *viz.*, the two forms of stacked-X and square planar states, and hence, it has been hypothesized that peak barriers between the states reduce due to the presence of peptides. However, even in hexapeptide bound conditions, the conformational diversity in the GC-based junction reduced with a greater preference toward the iso II state (Cannon et al., 2015). The time resolution of the sm-FRET method has successfully extracted the sequential intermediate dynamical state of the HJs bound to junction-resolving enzymes. Studies on the binding of sequence-independent and sequence-dependent enzymes with diverse types of stacked-X HJ segregated based on their branch migration properties have captured conformers of DNA promoted by the enzymes even for different ionic conditions. The protein-bound Holliday junctions have shown two FRET states corresponding to two alternative states differing in the coaxial pairing of the arms with a prominent shift compared to that of the unbound states. For all junction-resolving enzymes, *viz.*, bacteriophage T7 endonuclease I, prokaryotic RuvC, eukaryotic GEN1, and human endonuclease, hMus81-Eme1, a partially dissociated state is attained that synchronizes the process of conformer exchange for branch migration and eventually resolves the junction. The enzymes for branch migration and junction resolution bind together to the cross-over junction and form a ternary complex facilitating coordination between branch migration and junction resolution. This ternary complex is biased toward the promotion of branch migration (Zhou et al., 2019). The initial introduction of mechanical force through the onset of branch migration encourages the resolving enzyme to achieve a loosely bound conformation or partially dissociated (PD) state over complete dissociation. Although the force exerted has the potential for disruption of the heterologous junction, branch migration occurs only between identical sequences. Once the desired cleavage site is reached due to branch migration, the resolving enzyme switches to a fully bound conformation and performs the job of resolution (Figure 3D). The conformer exchange rates for hMus81-Eme1– bound junctions are the fastest, followed by GEN1- and RuvC-bound junctions, and T7 Endo I shows the slowest kinetics (Zhou et al., 2019).

### G-Quadruplex and I-Motif

**FIGURE 4:**
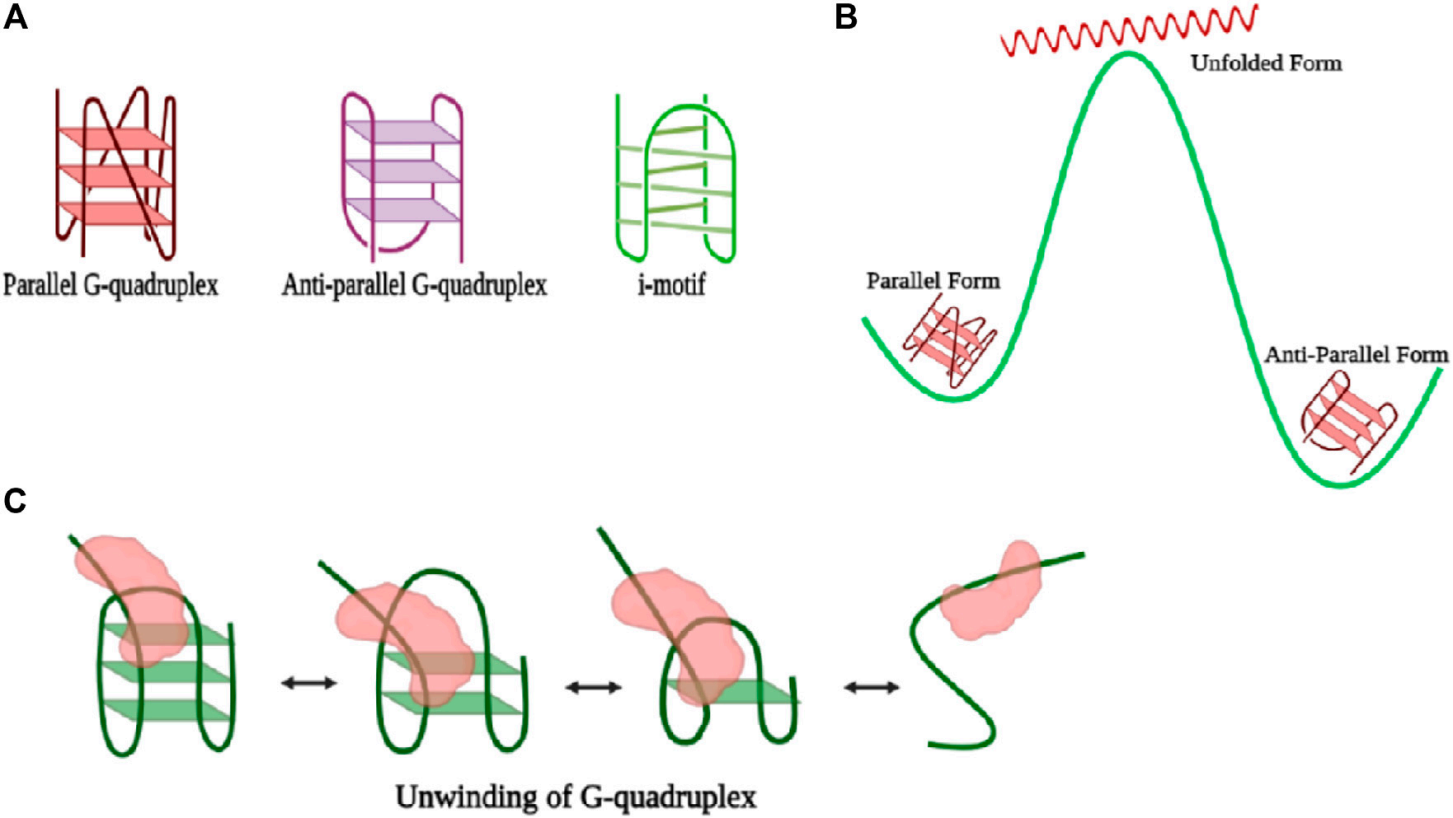
**(A)** Representation of different forms of the G-quadruplex. **(B)** Illustration of the energy landscape of various types of G-quadruplexes where the antiparallel form is the most stable; however, during conversion from the antiparallel to the parallel form, there is attainment of an intermediate high-energy unfolded state. **(C)** Schematic representation of unwinding of the G-quadruplex by a general helicase (colored in red). The folding and refolding process of the GQ motif is interconvertible (created with BioRender.com).

G-quadruplexes (GQ) are stable noncanonical secondary nucleic acid structures held together by G-G base pairs and are involved in transcription, translation, and replication processes. In consecutive guanine-rich single-stranded DNA or RNA regions, four guanine bases associate with each other through Hoogsteen type hydrogen bonds, giving rise to a horizontal square planar structure called the guanine tetrad where each corner of the tetrad is occupied by a guanine residue (Lipps and Rhodes, 2009). Two or more guanine tetrads stack over one another through π–π interactions between aromatic systems of G-quartets and finally give rise to a GQ structure promoting structural polymorphic properties and having high melting temperatures (Sen and Gilbert, 1988; Sundquist and Klug, 1989). Stabilization of the GQ is attributed through the stacking of the tetrad layers, hydration, and intercalation by monovalent cations either within the central cavity by relieving repulsion among the oxygen atoms of the tetrad or between the tetrad layers. Complementary to the G-rich sequences at any regulatory region such as telomeres, promoters (Sun and Hurley, 2009), introns, and UTRs, cytosine (C)-rich units occurring within the genomic DNA (Zeraati et al., 2018) that extrapolate themselves in a tetraplex structure and participate in gene regulatory functions are known as i-motifs (Figure 4A). Antiparallel cytosine-rich tracts intercalate to form a quadruple with two cytosines involved in intermolecular hydrogen bonding with one of the cytosines being hemi-protonated (Abou Assi et al., 2018). C-rich sequences present in the human telomeric region are analyzed by sm-FRET, and it is perceived that at low or acidic pH, 5.5 to be specific, they adopt a fully folded state. Upon introduction of neutral pH (6.5) in the medium, the dynamicity of the conformational changes is further enhanced, and dominance of the partially folded state takes place along with frequent inter-conversion to the unfolded and folded states. However, at high pH, the predominance of only the unfolded state is an indication of no formation of i-motifs at all (Megalathan et al., 2019).

Based on the orientation of the four strands, GQs are classified into two main types of structures, *viz.*, parallel and antiparallel forms, and have been captured in the presence of potassium (K^+^) and sodium (Na^+^) ions, respectively. When two of the strands in the tetrad possess an opposite polarity to the other two, the structure so formed is termed as antiparallel GQ, (Wang and Patel, 1993), whereas parallel quadruplex structures are formed if all four strands exhibit the same polarity in the same direction (Figure 4A) (Parkinson et al., 2002). Parallel and antiparallel GQs can be distinguished easily by circular dichroism (CD) spectroscopy, where the parallel form gives a peak at 260 nm and a valley at 240 nm and the antiparallel structure gives a positive peak at 292 nm and a negative peak at 260 nm (Paramasivan et al., 2007). Also, Raman spectroscopy is used to witness both the parallel and antiparallel forms in sodium and potassium solution, individually. The additional advantages of sm-FRET experimental analysis reveal the real-time temporal folding kinetics of the noncanonical G-quartet secondary structure and also captured the interconversion between the two forms. The kinetic rate of the transition from unfolded to folded states is highly dependent on the length and sequence of nucleotides present in the looping region. The higher the number of nucleotides in the looping region, the slower the dynamicity of the system, causing a reduced rate of transition and offering more persistence to short-looped DNA GQ (Miura et al., 1995). Also, sequences resembling human telomeric regions have faster folding rates than other GQ forming sequences which contribute toward the preservation of genome integrity by not allowing end-to-end nonspecific fusion (Tippana et al., 2014). Parallel and antiparallel GQ structures exist in equilibrium (Miura et al., 1995; Ren et al., 2002), whereas the antiparallel form is the dominant conformation with the lowest free energy in the presence of both Na^+^ and K^+^. Although free energy change between the conversion of antiparallel to parallel is lower than that of the conversion between the antiparallel and unfolded conformation, interconversion between the antiparallel and parallel states passes through unfolded or partially folded intermediates (Figure 4B) (Ying et al., 2003). The stability of parallel, antiparallel, hybrid, or loosely stable GQ structures is enhanced by several GQ binding proteins and GQ binding ligands (Brázda et al., 2014). When the central guanine of the G-quartet is converted to 8-oxo-guanine, full disruption of the GQ structure takes place in the telomeric region. Alternately, the introduction of an oxidative lesion to the thymine unit, *viz.*, Tg, brings different kinds of variations depending upon the structure of the GQ unit. For example, the structure of parallel and antiparallel GQ weakens, and the number of molecules exhibiting dynamic behavior increases (Lee et al., 2017).

Guanine-rich regions that are found mostly in human telomeres consist of a repetitive duplex sequence of TTAGGG (∼4–15 kb), followed by single-stranded 100–200 G-rich 3′ overhang, (Cimino-Reale et al., 2001) and attainment of GQ impedes the extension property of enzyme telomerase (Morin, 1989). Telomerases are primarily ribonucleoproteins where the RNA component directs the telomerase to the telomere unit and, utilizing the activity of the protein, the telomere overhang is extended in cancer cells, stem cells, and germline cells (Cano et al., 1999). Telomeres are also recognized by a six-membered shelterin protein complex that protects the 3′-overhang from DNA repair mechanisms and that also regulates the activity of telomerase. The subunits comprising the shelterin complex are TRF1, TRF2, POT1, RAP1, TIN2, and TPP1 (Xin et al., 2008). The role of POT1 is to protect the single-stranded telomeric DNA from nucleases (118) and to guide the suppression of the DNA repair pathway (Jones et al., 2016). Introduction of oxidative lesions in telomeric G-units controls the binding of POT1 protein. Damages that fully disrupt the G-quadruplex structure, for example, conversion of one of the guanines to 8-oxo-G, as has been verified through a significant FRET efficiency shift from that of the wild-type (from E_FRET_ = 0.85 to E_FRET_ = 0.53), efficiently facilitates POT1 binding. On the other hand, oxidation of thymine to thymine glycol (Tg) contributes toward less destabilization of the G-quadruplex, where the shift in FRET efficiency from the unmodified form is very meager, from 0.85 to only 0.8, and hence, does not promote efficient recruitment of POT1 (Lee et al., 2017). However, the introduction of base lesions and mutations in the nucleotides of the GQ forming sequence governs the accessibility of the telomerase to the telomeric DNA. For example, both 8-oxo-G and Tg enhance the binding and the activity of telomerase. Hence, unlike POT1, telomerase can overcome the barrier effect introduced by Tg by capturing the dynamically weak G-quadruplex structure (Lee et al., 2017).

**FIGURE 5:**
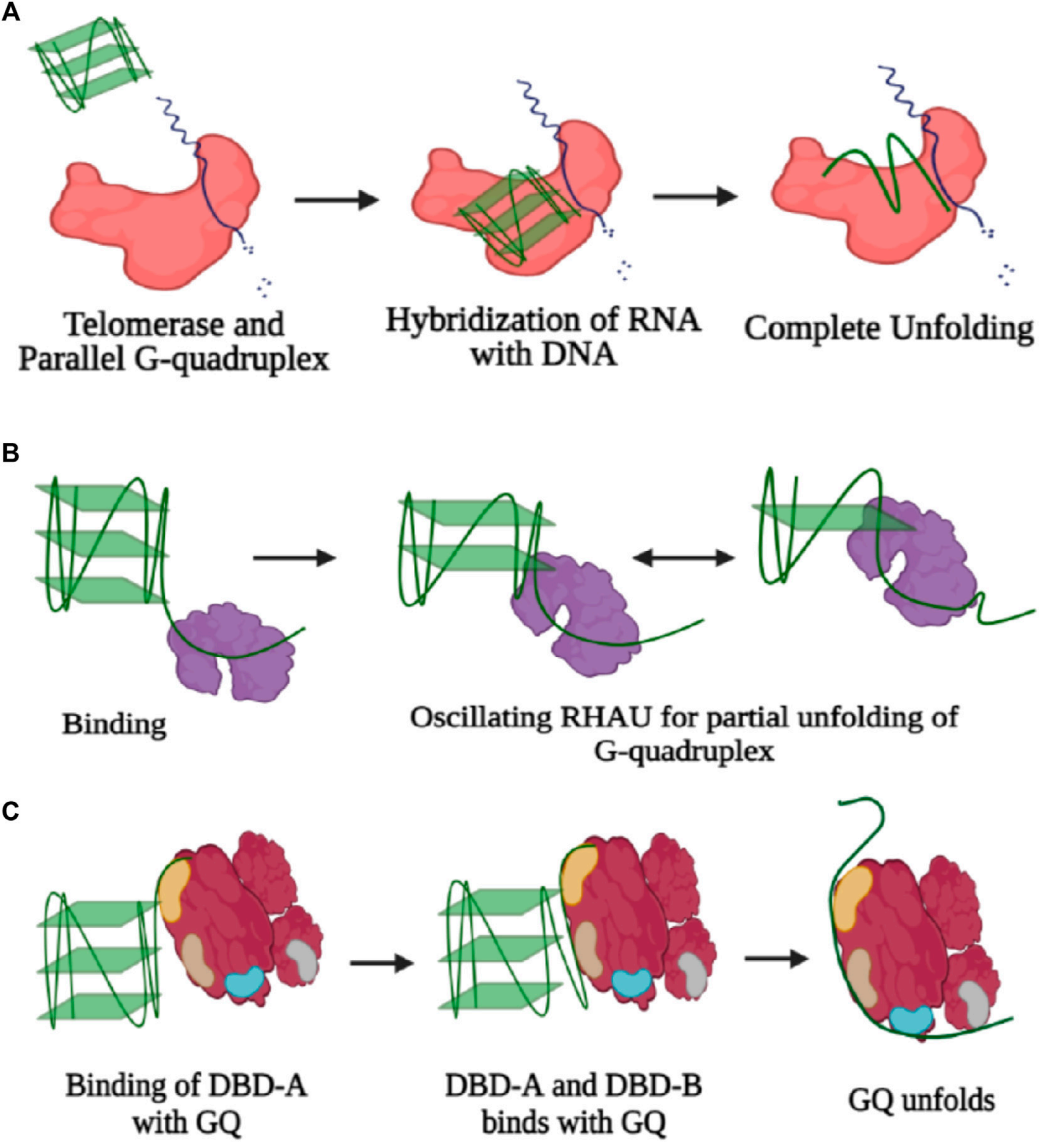
**(A)** Three-step unfolding of the parallel G-quadruplex by telomerase enzyme (colored in red). Initially, there is recognition and binding of GQ with telomerase, followed by partial unfolding *via* complementary binding of the RNA template with the G-quadruplex substrate. The 3′-end of the partial unwound GQ structure is extended by addition of single nucleotides, and eventually, there is complete disruption of the GQ structure. **(B)** Resolution of GQ by RHAU (colored in blue). Binding of RHAU occurs in the 3′ overhang, and further motility of the protein causes repetitive unfolding and refolding. **(C)** GQ unfolding by RPA. Initial recognition is through DBD-A and further contacts are established with DBD-B, followed by complete unfolding of the GQ structure through formation of contacts with other DBDs (created with BioRender.com).

Telomerases are also known to dislodge ligands from parallel structures of GQs to continue their activity without any hindrance, and the sm-FRET results from the study by Paudel et. al. (2020) have shown that parallel G4 stabilization with either small molecule ligands or by chemical modification does not always inhibit G4 unfolding and extension by telomerase. Depending on the position and type of base modification, telomerase activity is either inhibited or enhanced (Lee et al., 2020). Presence of GQ units in the promoter or untranslated regions (UTRs) of the genome regulates the process of transcription and gene expression. Furthermore, potential quadruplex-forming sites (PQSs) are also present in oncogene promoters, introns, exon boundaries, DNA replication origins, immunoglobin switch regions, and recombination sites (Maizels and Gray, 2013). In most cases, the presence of guanine-rich segments and their corresponding folding into the GQ structure challenges the machinery of replication, transcription, and translation since regulatory proteins need to exert a larger force to go ahead along with these structures (Rhodes and Lipps, 2015; Hänsel-Hertsch et al., 2017). The guanine repeats in the telomeric regions are very prone to oxidative damage and are hence termed as “hot zones.” Howsoever, parallel, antiparallel, or hybrid GQs all need to be resolved, at least partially if required, extended by telomerase (Moye et al., 2015). Resolution of even highly stable GQ in a conformation-specific manner is carried out by various helicases and to some extent, telomerase can also carry out the resolution of specifically telomeric GQ through a unique mechanism. Telomerase binds, unfolds, and extends intramolecular or intermolecular parallel GQ; however, antiparallel or hybrid conformation does not act as a substrate for telomerase (Hwang et al., 2014). The length of the telomeric overhang and its conformational dynamics greatly influence the loading and accessibility of telomerase toward GQ unfolding. As the length of the overhang increases to longer than four repeats, an exhibition of multiple conformational FRET states occurs in the GQ region; hence, an increment in dynamicity promotes better accessibility for several proteins, and telomerase is one of them. Howsoever, the recruitment of shelterin proteins such as POT1 and TPP1 does not get affected by the length of the overhang and loads on pre-folded telomeric DNA, followed by recruiting of telomerase (Hwang et al., 2014). Upon the binding of telomerase with the substrate irrespective of the process of catalytic activity, a partial unfolding of the G-quartet structure occurs through hybridization of the RNA unit of the telomerase with the telomeric DNA and that has been witnessed *via* a single-step drop in the FRET efficiency state when the labeled telomeric DNA is exposed to a high concentration of template RNA. Next, upon addition of nucleotides, there occurs a step-wise drop in the FRET state from 0.5 to 0.3 and further from 0.3 to 0.15. Thus, the results depict that the 3′ end of the partially unwound structure is extended *via* the addition of single nucleotides according to the RNA template until the terminal end, and finally, there is a complete unfolding of the GQ structure (Figure 5A). Basically, through conformational transition during translocation of the telomerase, there occur steric changes in the GQ structure which eventually lead to the unwinding of the G-quartet (Paudel et al., 2020).

Counteraction of the stable GQ structure by several helicases through the process of breakage of hydrogen bonds anchoring the structure of GQ is carried out *via* several mechanisms. Through single-molecule experiments, it has been possible to witness not only every step of the process but also dynamic fluctuations within each step of the GQ DNA. RNA helicase associated with AU elements, termed as RHAU, makes highly specific contact with the parallel GQ structure where one unit of the protein develops contact with the top of the parallel G-quartet structure, and the helicase domain engages itself in the unfolding of GQ. Also, RHAU requires a single-stranded DNA overhang to anchor itself for the endorsement of unwinding activity (Figure 5B) (Tippana et al., 2016). However, in the case of Bloom’s helicase, there is no discrimination in the selection of GQ structures, and hence, binding occurs equivocally with all kinds of GQ structures, that too in an ATP-independent manner. The most specificity for telomeric GQ sequences has been observed in the case of the Werner helicase (WRN), and also, there is essential involvement of ATP in the unwinding activity, and thus, the activity rate is 5–10 times faster than those of the BLM and RHAU helicases (Tippana et al., 2016). Furthermore, through the course of studies, it has been verified that like the activity of many helicases, GQ unwinding is also carried out reversibly, and re-annealing of the structure occurs after a certain limit of unwinding (Figure 4C). The phenomenon of the repetitive cycle of unfolding and refolding of secondary structures is well conserved within several helicases. This process has proved itself highly efficient for protecting ssDNA or ssRNA (single-stranded RNA), which are prone to engagement in several unintended processes. Also, repetitive motion of the helicase enzyme ensures that a single enzyme is efficiently modulated to engross itself in multiple rounds of the unwinding procedure, ensuring saving of energy for the system (Wu et al., 2017). Nevertheless, the unfolding rate for RHAU is slower than the refolding rate and over that only partial disruption of the structure since RHAU encourages the process of C-strand fill-in. Hence, confiscation of the G-rich unit through complementary C-rich strands forbids unwanted exertion of other cellular machinery units. Correspondingly, RHAU can also displace GQ-stabilizing chemical ligands *via* its unwinding activity, for example, BRACO-19 (Tippana et al., 2016). Eukaryotic single-stranded binding protein analog RPA also participates in the resolution of GQ structures not only in telomeric regions but also in all potential GQ forming sites (PQSs). RPA requires a longer single-stranded nucleotide region to bind and mediates destabilization of the GQ in a two-step–dependent fashion. Initially, contact is developed between one of the DNA binding domains (DBD-A) of RPA with the single-stranded overhang, followed by recruitment of another unit, DBD-B, and together these two units initiate the unfolding mechanism of GQ. Once there is a kick-start in the process of GQ unfolding, the other domains of the protein establish their contacts with the single-stranded DNA (Figure 5C) (Ray et al., 2013). Another single-stranded DNA (ssDNA) binding protein, human CST resembling RPA carries out the process of GQ resolution. The rate of GQ unfolding by CST is more than even shelter in proteins, like POT1. Human CST binds in a region adjacent to the dsDNA-ssDNA hybrid form and introduces dynamicity to the whole complex other than promoting the opening of the GQ structure. This induced dynamic nature contributes toward the recruitment of DNA polymerase and replisome machinery promoting the process of C-strand fill-in (Bhattacharjee et al., 2017). Cooperation of RPA with one of the RecQ family helicases, RECQ5 enhances the GQ destabilizing property of RECQ5 by diminishing the strand-annealing property of RECQ5. Otherwise, while unaccompanied by RPA even in the presence of an adequate concentration of ATP, RECQ5 cannot efficiently unfold GQ in the presence of KCl where GQ is known to be the most stable. However, the rate of unfolding increases when GQ is supplemented with weak stabilizers such as NaCl or TBA (thrombin binding aptamers) (Budhathoki et al., 2016). Also, GQ stabilizing ligands cause a decrement in the activity of GQ unfolding by helicases (Budhathoki et al., 2016). Sensitivity of GQ unfolding activity to the degree of stability of the GQ structure is valid for several other helicases, for example, RNA helicase DHX36 (RHAU). Also, DHX36 was found to induce conformational changes in the GQ structure in an ATP-independent manner, and hence, it was deduced that ATP is required only in the step of the release of DNA from the helicase since the partial oscillation of the helicase along the DNA for repetitive unfolding activity is ATP independent (Figure 5B) (Chen et al., 2018). However, the activity of the other RecQ family helicase, the Bloom helicase (BLM), cannot proceed with the course of the unfolding of the GQ structure without ATP or a non-hydrolyzable analog of ATP. BLM requires a 3′-overhang to get loaded and starts destabilizing GQ in three steps. In the very first step, there is an active translocation of BLM in the 3′-5′ direction along with the unfolding of the GQ structure. Finally, after complete unwinding, there occurs release of BLM from the single-stranded DNA (ssDNA) and immediate refolding of the ssDNA into GQ (Wu et al., 2015).

### Limitations

The exertion and execution of sm-FRET experiments is also associated with certain practical and conceptual challenges that bring limitation to the system, too. One of the considerable constraints is the limited distance range (∼3–10 nm), where information can be gained through FRET and any slippage beyond cannot trigger FRET (Banterle and Lemke, 2016). Additionally, a huge compromise with the signal-to-noise ratio interferes with the measurement in a microscopic system, as the acquisition of photons emitted by a fluorophore is reduced to 10–15% of the total number (34, 39). The intrinsic properties of the dyes, for example, photobleaching and quantum yield, limit the time and data quality of monitoring the dynamics of the biomolecule. Although sm-FRET has proven itself a fine tool for *in vitro* studies, the intrinsic auto-fluorescence of the cellular system and precise delivery of the sample of interest inside the cell are truly challenging for *in vivo* measurements (Bacia and Schwille, 2003). Nevertheless, there is continual improvisation going on in this field to overcome these limitations. Replacement of the TIRFM set-up with light sheet microscopy (55) may solve the issue of the low signal-to-noise ratio and photobleaching and will bring enhanced precision toward monitoring *in vivo* samples.

### Future Perspectives

Monitoring of conformational and structural properties by single-molecule studies can also be further expanded for exploration of dynamic properties of several other conventional and unconventional DNA secondary structures that either are a part of an *in vivo* process or can be synthesized artificially to be utilized as nano-devices (Krishnan and Simmel, 2011). These structures may include Z-DNA, cruciform DNA, and triplex-forming oligonucleotides, to name a few. The introduction of negative supercoiling stabilizes the formation of a left-handed DNA motif, termed as Z-DNA under the physiological salt condition (Rahmouni and Wells, 1989) that eventually relieves transcriptional torsional stress, although their presence in the transcription start site causes genome instability (Zhao et al., 2010). Single-stranded intermediates also lead to the formation of cruciform DNA that is formed when two continuous DNA strands reorganize themselves into a four-way structure, and their location is primarily in the replication origins, promoter region, and breakpoint junctions (Pearson et al., 1996; Lobachev et al., 2007). Additionally, structural perturbation in the double-stranded structure of DNA leads to the accommodation of one more single strand and that is exemplified in the form of a triple helix structure, a purine-based intermediate strand with two pyrimidine strands on both sides. If the third pyrimidine strand is parallel, both Hoogsteen and Watson–Crick bonds are involved in the triplex formation. However, if the third strand of the triplex is antiparallel with respect to the duplex, this involves reverse Hoogsteen base-pairing and even develops at physiological pH (Manor et al., 1988; Jain et al., 2008). All these structures carrying both structural and functional diversity provide an immense opportunity for their detailed study. With improved understanding of functional and dynamical aspects of these fundamental biopolymers, the path of applied science toward bioengineering, therapeutics, and the diagnostic field would become smoother and more accessible.

### Summary

In this review, we have presented a summary of the conformational dynamics of the important DNA secondary structures extracted at the molecular level by sm-FRET. The knowledge about the structure of biological macromolecules at atomic resolution gained through X-ray crystallography, NMR, electron microscopy, or computation approaches has revolutionized their visualization with complete accuracy and has uncovered several hidden complexities. Yet, the details visualized and extrapolated are of the state having the lowest energy and the maximum population. The information about the wiggling of the biomolecules in the lowest energy state itself and its transition through a high-energy barrier followed by downhill motion toward a more stable state are very crucial for the exploration of functional and regulatory aspects (Austin et al., 1975; Kern, 2021). However, in the structural analysis approach, the dynamicity and transition between several states are missing. The emergence of the technique of FRET at the single-molecule level made it possible to map the functional properties with conformational heterogeneities. Through sm-FRET, it is not only possible to witness the dynamical conformational states acquired by a biomolecule with all degrees of conformational and functional heterogeneity present in it but also we can deduce the quantitative kinetic details (McKinney et al., 2006; Lee, 2009) at a very high temporal resolution, to date, the highest being reported at 1 ms. Hence, for the secondary structures of DNA, sm-FRET has paved the way to determine the conformation where the molecule stays for the maximum amount of time with its stability and, at the same time, the frequency of structurally allowed transitions along with their rates over a range of timescales (seconds to minutes) until the molecules photobleach, and that makes sm-FRET an efficient tool for the exploration of ‘dynamic structural biology’ (Lerner et al., 2021). The trajectory of every *i*th molecule can be recorded, analyzed, and its dwell time at every dynamical state can be obtained. Besides, integration of sm-FRET with other single-molecule techniques such as optical tweezers, AFM (atomic force microscopy), and fluorescence correlation spectroscopy (FCS) paves the way toward more parameters with promising results.
